# Impact of Chlorogenic Acid on Peripheral Blood Mononuclear Cell Proliferation, Oxidative Stress, and Inflammatory Responses in Racehorses during Exercise

**DOI:** 10.3390/antiox12111924

**Published:** 2023-10-28

**Authors:** Izabela Dąbrowska, Jowita Grzędzicka, Adrianna Niedzielska, Olga Witkowska-Piłaszewicz

**Affiliations:** Department of Large Animal Diseases and Clinic, Institute of Veterinary Medicine, Warsaw University of Life Sciences, 02-787 Warsaw, Poland

**Keywords:** green coffee extract, chlorogenic acid, lymphocytes, anti-inflammatory cytokines, racing, natural supplements, exercise, monocytes, sport, oxidative stress

## Abstract

Green coffee extract is currently of great interest to researchers due to its high concentration of chlorogenic acid (CGA) and its potential health benefits. CGA constitutes 6 to 10% of the dry weight of the extract and, due to its anti-inflammatory properties, is a promising natural supplement and agent with therapeutic applications. The purpose of our study was to discover the effects of CGA on peripheral blood mononuclear cell proliferation, and the production of pro- and anti-inflammatory cytokines as well as reactive oxidative species (ROS) in horses during exercise. According to the findings, CGA can affect the proliferation of T helper cells. In addition, at a dose of 50 g/mL, CGA increased the activation of CD4+FoxP3+ and CD8+FoxP3+ regulatory cells. Physical activity decreases ROS production in CD5+ monocytes, but this effect depends on the concentration of CGA, and the effect of exercise on oxidative stress was lower in CD14+ than in CD5+ cells. Regardless of CGA content, CGA significantly increased the release of the anti-inflammatory cytokine IL-10. Moreover, the production of IL-17 was greater in cells treated with 50 g/mL of CGA from beginners compared to the control and advanced groups of horses. Our findings suggest that CGA may have immune-enhancing properties. This opens new avenues of research into the mechanisms of action of CGA and possible applications in prevention and health promotion in sport animals.

## 1. Introduction

There has been a recent increase in the social awareness and understanding of the health-promoting properties of food. Eating less processed and/or more additive-free natural foods seems to be particularly common. Among unprocessed beverages, apart from water, coffee is the most popular drink in the world, with consumption increasing by as much as 3.3% and global sales of 170.3 million 60 kg bags in 2021/2022 compared to 2020/2021 [1]. The most common commercially available coffee is *Coffea arabica* L., *C. canephora Pierre ex A. Froehner* (also known as Robusta coffee), and *C. liberica Bull.* ex Hiern [2]. The chemical composition of roasted coffee varies depending on the kind. Coffee acids have a considerable impact on taste and aroma as well as serving as flavor precursors [3,4]. Caffeic acids are classified as organic acids (OA), chlorogenic acids (CGA), and inorganic acids like phosphoric acid [5], with CGA receiving the most interest in recent years. CGA is not only a natural diet supplement [6,7], studies indicate that it is also clinically safe regarding side effects, and its long-term administration has not caused changes in serum biochemical variables, including no significant changes in serum iron, magnesium, copper, zinc or vitamin B1 [8].

Chlorogenic acid (CGA) belongs to the group of hydroxycinnamates, esters of cinnamic acid derivatives with quinic acid, which are widely distributed in plant materials such as apples [9], pineapple [10], blueberries [11] and coffee seeds, in which the concentration of CGA is as much as 6–10% of dry matter, leading such plants to contain its highest concentration [12]. 5-O-caffeoylquinic acid (5-CQA) is the most prevalent type and accounts for 76–84% of all CGAs [13], but monoesters from caffeic acids (caffeolic acids, CQA), p-coumaric acid (p-coumaroylquinic acids, p-CoQA), and ferulic acid (feruloylquinic acids, FQA) are also prevalent [14]. CGA is proven to possess antioxidant [9,15], anti-inflammatory [16,17,18,19,20], antibacterial [21,22,23,24,25] and anticancer [26,27,28,29,30] effects both in humans and animals. It was proven that CGA at various doses (0, 2, 5, and 20 lM) reduces the production of pro-inflammatory cytokines like IL-1, TNF-α, and IL-6 in a dose-dependent manner in mouse macrophage cell line RAW 264.7 and BV2 microglial cells stimulated by lipopolysaccharide (LPS) [19]. Another study conducted on human peripheral blood mononuclear cells (PBMSs) proved that CGA can reduce the production of IL-1b, IL-6, TNFa, and IFNg at 86%, 90%, 84%, and 95%, respectively, at 200 mg/mL dose while levels of MCP-1, MIP-1a, and MIP-1b were decreased by 99%, 77%, and 91%, respectively [16]. Worth mentioning is that CGA is not only safe for the liver and plasma [6] but systematic consumption also leads to lower fasting plasma glucose by reducing its absorption of glycosylated hemoglobin and insulin levels by up to 6.9% [31,32,33,34,35].

The promotion of the anti-inflammatory process shows promise as an effective therapeutic target for treating traumatic muscle injuries [36]. Studies reveal that during physical activity in both race and endurance horses, muscle cramps accompanying muscle fiber damage initiate an exercise-induced inflammatory response [37,38,39]. It has been found that physical activity such as an ultramarathon stimulates the release of IL-6, IL-10, and IL-1ra [40]. It is crucial since they can suppress the production of IL-1-type cytokines and regulate the early inflammatory response to exercise in both people and horses. IL-6 release regulates metabolism and is vital in boosting satellite cell renewal and proliferation. Satellite cells, in turn, can influence muscle regeneration by secreting platelet-derived growth factor (PDGF) and IL-6, both of which encourage cell proliferation and differentiation in the healing area. The following findings suggest that extensive endurance training in horses leads to the development of a reduced inflammatory capacity, as seen by a drop in type 1 pro-inflammatory cytokine concentrations over time, and maybe the development of an anti-inflammatory condition [37]. The horse’s adaptability to training, particularly the first three months of regular development, appears to be crucial to the adaptation process, with the main anti-inflammatory changes occurring in the second and third months, and the cumulative effect of the training process may be a consideration. A similar finding was confirmed in racehorses. It has been demonstrated that IL-6, IL-13, IL-10, or IL-1ra can mediate the protective long-term anti-inflammatory benefits of exercise by increasing the anti-inflammatory response [38].

Racehorses, due to their intensive training regimen are constantly exposed to physical stress stimuli. Intensive physical exercise is an acute mechanical and metabolic load that induces a wide systematic response to preserve homeostasis. It has been widely described that one of the consequences of physical exercise is changes in immunological parameters [41,42,43]. The inflammatory signaling cascade triggered by working muscle involves changes in peripheral blood cell numbers, granulocyte activity, NK cell cytotoxic activity, lymphocyte proliferation, and cytokine levels in plasma among others [44,45,46,47]. The inflammatory process is crucial for musculoskeletal system remodeling; however, an unbalanced inflammatory response may lead to tissue destruction [48]. The hunt for alternative remedies that can balance this inflammatory response may be critical to improving horse health and sports performance. Supplements that may have a positive impact on horse athletes’ performance are becoming more and more popular nowadays, especially those of natural origin. Plant-origin immunomodulators such as CGA are used in animal nutrition. However, there is a lack of studies concerning the CGA influence on horses. In addition, it is possible to use decaffeinated green coffee seeds containing CGA which is very important especially for athletes to avoid doping accusations. Thus, the goal of this study is to evaluate how natural and safe components such as CGA impact the inflammatory reaction and oxidative stress triggered by physical exertion in horses.

## 2. Materials and Methods

### 2.1. Animals and Blood Sampling

The investigation encompassed the impact of CGA on immune cells in racehorses at different fitness levels. A total of 29 healthy racehorses, aged 2–7 years, were included in the study, with 15 males and 14 females. The horses were divided into two groups: the experienced group (advanced group), consisting of 7 Thoroughbreds (average age: 3 ± 0.37 years) and 7 Arabians (average age: 6 ± 1.29) with a history of good performance in the previous training seasons, and the inexperienced group (beginners), comprising 9 Thoroughbreds (average age: 2 ± 0) and 6 Arabians (average age: 4 ± 0.82) at the beginning of their race training career. The good performance was determined based on the history of the previous training season. The inexperienced group consisted of horses during their first training season.

Both groups were subjected to the same environmental conditions and training regimen, considering their respective fitness levels. All horses were stabled and trained by a single trainer. Prior to the study, a veterinary practitioner conducted clinical examinations, including assessment of heart rate, mucous membrane color and moisture, capillary refill time, and dehydration (measured by the time it takes for a pinched skin to fold over the point of the shoulder and flatten). Additionally, basic blood hematological and biochemical tests were performed. No clinical symptoms of diseases were observed in any of the horses.

The exercise session took place on an 800 m track, with all horses running at a speed of approximately 800 m/min. Blood samples were collected before, immediately after, and thirty minutes after the exercise session according to the routine protocol for fitness monitoring from all horses through jugular venipuncture, using K2EDTA tubes within a BD vacutainer system (BD Vacutainer^®^, Franklin Lakes, NJ, USA), for the isolation of peripheral blood mononuclear cells (PBMCs). For the purposes of this study, only the samples taken before and immediately after the exercise were analyzed. Only excess peripheral blood collected for routine diagnostic tests was used for this study. It is important to note that all sampling procedures adhered to the standard veterinary diagnostic protocol and were performed in compliance with Polish legal regulations and the European directive EU/2010/63. Ethical approval from the Local Commission for Ethics in Animal Experiments was not required for this study.

### 2.2. Chlorogenic Acid

Chlorogenic acid (CGA) obtained from Sigma-Aldrich (Sigma-Aldrich, Saint Louis, MO, USA) was solubilized in phosphate-buffered saline (Life Technologies, Bleiswijk, The Netherlands). Cells were cultured with 15 µg/mL and 50 µg/mL CGA which was used based on previous human, laboratory animal, and cell culture publications [8,9,10,11,12,13,14,15,16,17,18,19,20,21,22,23,24,25,26,27,28,29,30,31,32]. For the control treatments, the same procedures were followed, but without the addition of CGA. Samples from corresponding horses were used for the control treatments.

### 2.3. Cell Isolation and Culture

PBMCs were obtained from the K2EDTA tube blood of all horses using density gradient centrifugation (SepMate™-Lymphoprep™ System, Cologne, Germany). The cells were centrifuged at 1200× *g* for 10 min following the manufacturer’s instructions. The cells were washed twice in 2% BSA and frozen in 10% DMSO in heat-inactivated horse serum (IHS) at −80 °C for further analysis.

Subsequently, the cells were refrozen, washed twice in 2% BSA and cell cultures were established using RPMI 1640 Medium with GlutaMAX™ (Gibco, Life Technologies, Bleiswijk, The Netherlands), supplemented with 10% IHS, penicillin (100 IU/mL), streptomycin (100 μg/mL), nonessential amino acids (1%), MEM vitamins (100 μM), sodium pyruvate (1 mM), and amphotericin B (1 μg/mL) (Gibco™, Life Technologies, Bleiswijk, The Netherlands).

PBMCs were placed in a 96-well flat-bottom plate (353072, Falcon, BD, Franklin Lakes, NJ, USA) at a concentration of 2 × 10^5^ cells in 200 μL of the culture medium per well. PBMCs were cultured in the absence or presence of phytohemagglutinin (PHA) (Sigma-Aldrich, St. Louis, MO, USA; 5 μg/mL). After 24 h, the cells were washed, and recombinant equine IL-2 (R&D Systems, Abingdon, UK; 100 U/mL) was added. The cells were then incubated for an additional 3 days at 37 °C with 5% CO_2_.

### 2.4. Cell Staining

Samples stained with CellTrace™ Violet Cell Proliferation Kit (Life Technologies, Bleiswijk, The Netherlands) prior to culturing were used in a cell proliferation assay. The procedure was conducted using the manufacturer’s instructions.

After 4 days of culture, the production of reactive oxygen species (ROS) by isolated PBMCs was assessed using the CellRox (CR) Deep Red Assay Kit (Life Technologies, Paisley, Scotland), following the manufacturer’s protocol.

To analyze lymphocytes, non-adherent cells were specifically collected (these were also used for cell proliferation assessment). Equine-specific antibodies or antibodies with documented cross-reactivity (as indicated in Table 1) were utilized to evaluate the surface marker expression on PBMCs. To minimize nonspecific antibody binding, 10% BSA was used for blocking (15 min at 4 °C) prior to staining with antibodies. The cells were incubated with antibodies in eBioscience™ Flow Cytometry Staining Buffer (Life Technologies, Bleiswijk, The Netherlands) in the dark for 20 min at 4 °C. Subsequently, the cells were washed twice with 2% BSA, resuspended in 200 μL of flow cytometry staining buffer, and immediately analyzed using a cytometer.

For FoxP3 staining, the eBioscience™ FoxP3/Transcription Factor Staining Buffer Set (Life Technologies, Bleiswijk, The Netherlands) was utilized following the manufacturer’s protocol.

### 2.5. Flow Cytometry Analysis

A similar gating strategy utilized in this study was previously described in a previous publication [47]. To ensure the analysis of single cells, doublets were excluded by setting a gate based on the FSC-area (FSC-A) vs. FSC-high (FSC-H) dot plot. Cell proliferation calculations were performed exclusively on singlet cells. The gate specifically included lymphocytes, and further analyses were conducted on CD4+, CD8+, and FoxP3+ cell populations. In the case of the second sample, the gate encompassed CD5+, CD14+, MHCII+ cells, and the median fluorescence intensity (MFI) of reactive oxygen species (ROS) was determined within that specific cell population.

Flow cytometric analysis was carried out using a FACSCanto II flow cytometer and FlowJo™ version 10.9 software (Becton, Dickinson, NJ, USA). A total of 10.000 cells from each sample were acquired for analysis.

### 2.6. ELISA

Cytokine concentrations (IL-1β, IL-4, IL-8, IL-10, IL-17, INF-γ, and TNF-α) were assessed in post-exercise samples treated by CGA in different doses using immunoenzymatic commercial assays specifically designed for equine species (ELK Biotechnology, Wuhan, China). Absorbance readings were obtained with a Multiscan Reader (Labsystem, Helsinki, Finland) and analyzed using Genesis V 3.00 software.

### 2.7. Statistical Analysis

Statistical analysis was conducted using the OriginPro 2022 statistics package (OriginLab Corporation, Northampton, MA, USA). A one-way repeated measures ANOVA was utilized to assess the impact of CGA on horses’ PBMCs across three different concentrations. To compare individual groups, a post-hoc pairwise comparison was performed with the Bonferroni test. Significance was determined at a *p*-value of 0.05.

In cases where the data violated the normality assumption, a non-parametric Friedman ANOVA test was employed. A post-hoc analysis for the aforementioned test involved the Wilcoxon–Nemenyi–McDonald–Thompson test. Significance was evaluated at a 0.05 level.

Prior to the analysis, outlier detection was conducted using Grubbs’ test or based on linear regression to ensure data integrity.

## 3. Results

### 3.1. Chlorogenic Acid Effect on Lymphocyte Phenotype and Proliferation

**Figure 1:**
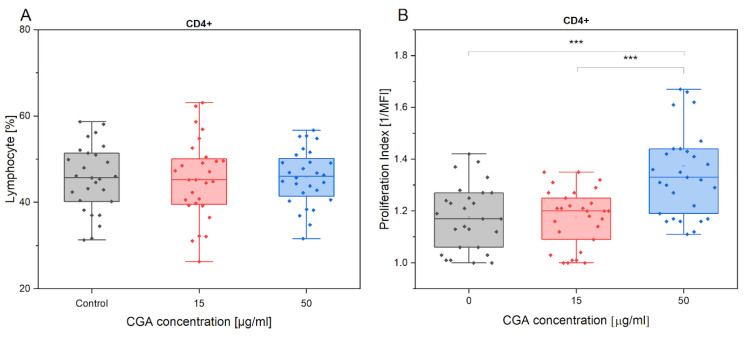
Presentation of CD4+ % (**A**) and its proliferation index (**B**) variations in the absence and presence of CGA at 15 µg/mL and 50 µg/mL concentrations. Each dot represents one horse sample in a particular treatment condition, and means ± SEM (standard error of the mean) are presented. Significance levels are: *** *p* < 0.001.

The lack of statistical significance indicated CGA had no effect on percentage of CD4+ cells (Figure 1A). Although CGA left the CD4+ percentage unaffected, the compound significantly influenced the proliferation of helper T cells. The highest concentration used (50 µg/mL) enhanced the activity of CD4+ cells in comparison to the control treatment (*p* = 6.6 × 10^−6^) (Figure 1B). Moreover, there was a significant difference when comparing it to the 15 µg/mL CGA treatment (*p* = 5.6 × 10^−7^). Regardless of CGA treatment, the CD4+ % remained unchanged in response to exercise. Furthermore, considering the horses’ level of advancement, the percentage of CD4+ cells showed no variation following exercise. (Figure S1).

**Figure 2:**
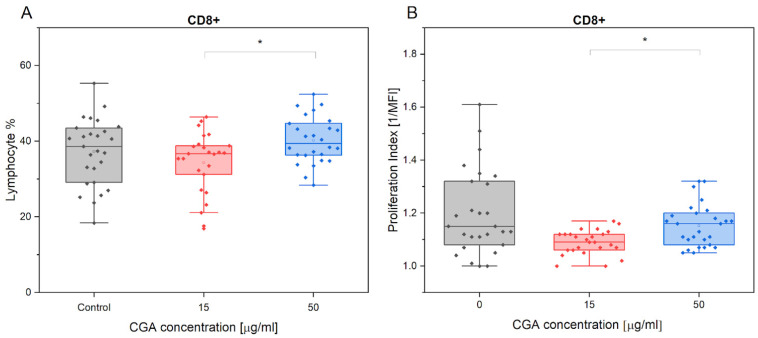
CD8+ % (**A**) and proliferation index (**B**) variations in the absence and presence of CGA at 15 µg/mL and 50 µg/mL concentrations. Each dot represents one horse sample in a particular treatment condition, and means ± SEM (standard error of the mean) are presented. Significance levels are: * *p* < 0.05.

Statistically significant findings were only observed between two of the CGA concentrations used (*p* = 0.017). Specifically, CGA showed no significant effect on the % of CD8+ cells compared to the control treatment (Figure 2A). Similar results were also confirmed for the measured proliferation (Figure 2B).

Similar to the results in the case of CD4+ lymphocytes, exercise established no effect on CD8+ lymphocyte count regardless of CGA treatment (Figure S2).

### 3.2. Chlorogenic Acid Effect on T Regulatory Cells

**Figure 3:**
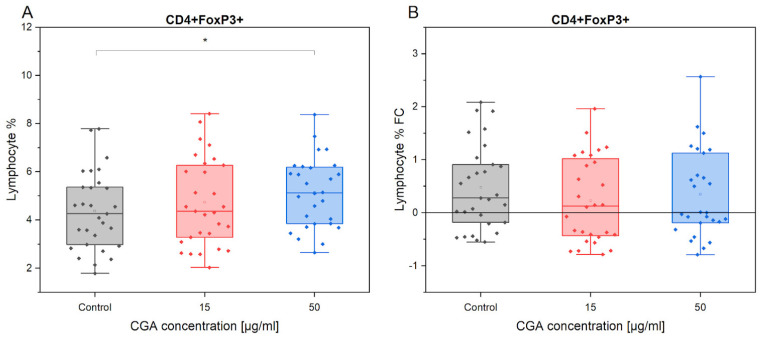
Presentation of CD4+FoxP3+ count variations in the absence and presence of CGA at 15 µg/mL and 50 µg/mL concentrations (**A**), and its fold change in response to exercise (**B**). Each dot represents one horse sample in a particular treatment condition, and means ± SEM (standard error of the mean) are presented. Significance levels are: * *p* < 0.05.

The analysis of CD4+FoxP3+ cell % variation between the absence of CGA treatment and 50 µg/mL CGA treatment revealed a statistically significant increase in regulatory lymphocytes after CGA treatment (*p* = 0.02558) (Figure 3). The observed difference could imply that CGA at 50 µg/mL plays a role in modulating the number of regulatory CD4+FoxP3+ T cells.

**Figure 4:**
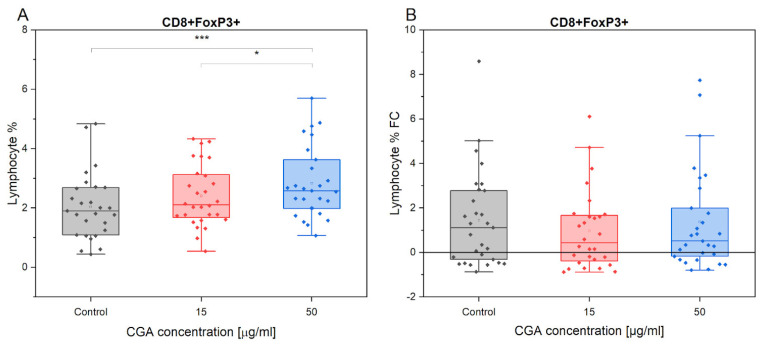
Presentation of CD4+FoxP3+ count variations (**A**), and its fold change in response to exercise (**B**) in the absence and presence of CGA at 15 µg/mL and 50 µg/mL concentrations. Each dot represents one horse sample in a particular treatment condition, and means ± SEM (standard error of the mean) are presented. Significance levels are: * *p* < 0.05 and *** *p* < 0.001.

A statistically significant increase in CD8+FoxP3+ cell production was observed in response to CGA treatment at a concentration of 50 µg/mL compared to the control treatment (*p* = 0.00015) (Figure 4). However, treatment with CGA at a dose of 15 µg/mL did not significantly stimulate regulatory lymphocytes compared to the control treatment. Notably, a significant difference was observed when comparing the highest concentration (50 µg/mL) to the 15 µg/mL treatment, confirming a strong stimulation of CD8+FoxP3+ (*p* = 0.047). These findings suggest that CGA may stimulate the polarization of CD8+FoxP3+ regulatory lymphocytes specifically at the concentration of 50 µg/mL.

The administration of CGA treatment led to an increase in the T reg population in response to exercise. While the control treatment (FC = 1.19) showed the most significant change. However, there were no significant differences observed in the magnitude of this change between different treatment conditions in response to exercise.

### 3.3. Chlorogenic Acid Effect on Monocyte Phenotype

According to the established criteria [47], changes in the proportion of CD14−MHCII+ non-classical, CD14+MHCII+ intermediate, and CD14+MHCII− classical monocyte cells observed in both inexperienced and experienced equine subjects following treatment with varying concentrations of CGA, as well as the FC of pre- to post-exercise did not show statistically significant findings (Figures S4–S7).

However, it can be noted that overall, the proportion of CD14+MHCII− classical monocytes prevails in the investigated horse PBMCs, while CD14−MHCII+ non-classical monocytes are the least abundant (Figure S3).

### 3.4. Chlorogenic Acid Effect on ROS Production in Monocytes and Lymphocytes

**Figure 5:**
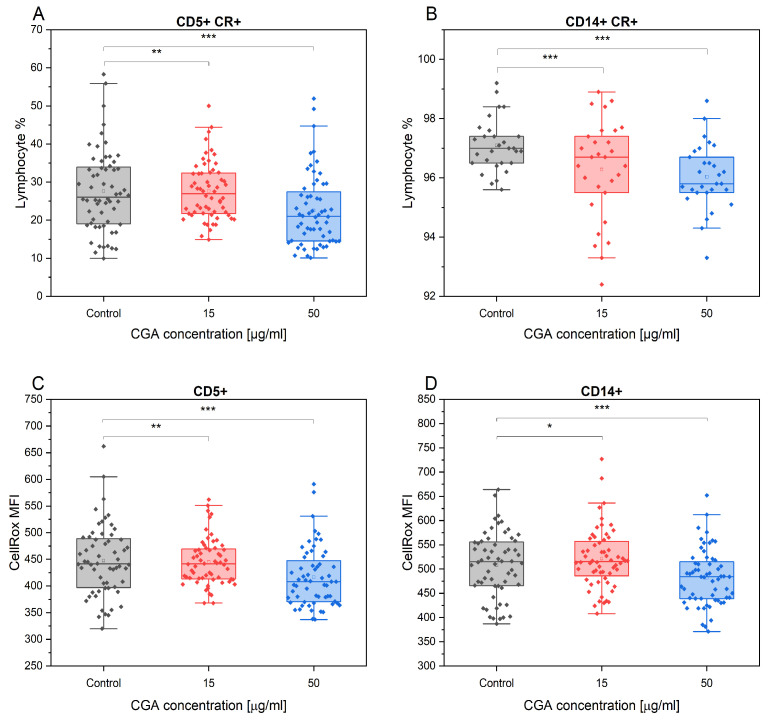
The CD5+ (**A**) and CD14+ (**B**) percentage gated from total lymphocytes/monocytes positive for CellRox fluorescence and the mean fluorescence intensity (MFI) of CellRox (ROS accumulation quantity) in each subtype (**C**,**D**) in response to the absence and presence of CGA at 15 µg/mL and 50 µg/mL concentrations. Each dot represents one individual horse’s sample in a particular treatment condition, and means ± SEM (standard error of the mean) are presented. Significance levels are * *p* < 0.05, ** *p* < 0.01 and *** *p* < 0.001.

CGA exhibited a notable reduction in percentage of the CD5+ T lymphocytes and CD14+ monocytes that produced ROS, as assessed by the CellRox assay. This effect was most pronounced at the highest concentration of CGA tested, compared to the control treatment (CD5+: *p* = 3.1 × 10^−5^, CD14+: *p* = 1.34 × 10^−6^). However, the observed effect at 50 µg/mL did not significantly differ from the cells treated with 15 µg/mL (Figure 5A,B). Furthermore, CGA treatment significantly reduced the overall ROS accumulation (MFI) in the CD5+ and CD14+ cell subtypes in comparison to the control treatment. The extent of this effect depended on the concentration of the compound, and it is the highest for 50 µg/mL (CD5+: *p* = 7.88 × 10^−5^; CD14+: *p* = 1.34 × 10^−6^) (Figure 5C,D).

Notably, exercise diminished the CD5+ lymphocyte proportion identified as ROS source (FC = −0.4). This effect was clearly observed under CGA treatment conditions. However, at 15 µg/mL CGA, CD5+ lymphocyte proportion that produced ROS seemed to be reduced to a significantly lower extent in comparison to the control (*p* = 4.8 × 10^−6^) and 50 µg/mL treatment (*p* = 6.1 × 10^−7^).

**Figure 6:**
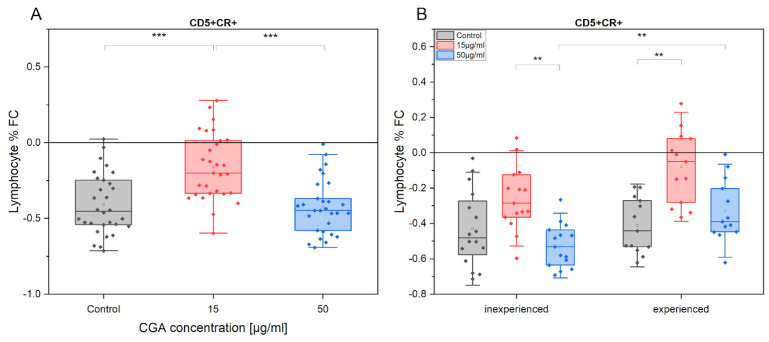
The fold change (FC) of CR (CellRox) intensity of CD5+ percentage gated from total lymphocytes/monocytes in response to exercise (**A**) in the absence and presence of CGA at 15 µg/mL and 50 µg/mL concentrations and at different horses’ advancement level (inexperienced, experienced) (**B**). Each dot represents one individual horse sample in a particular treatment condition, and means ± SEM (standard error of the mean) are presented. Significance levels are: ** *p* < 0.01, and *** *p* < 0.001.

The tendency of these changes was observed in both horses’ advancement level groups. The average fold change (FC) of ROS production in the absence of CGA was comparable between the inexperienced (FC = −0.42) and experienced (FC = 0.41) groups (Figure 6A). However, following CGA treatment, the oxidative stress response was different. Inexperienced horses’ samples demonstrated a greater reduction in oxidative stress after training when treated with 50 µg/mL of CGA, compared to the experienced group (*p* = 0.003) (Figure 6B).

**Figure 7:**
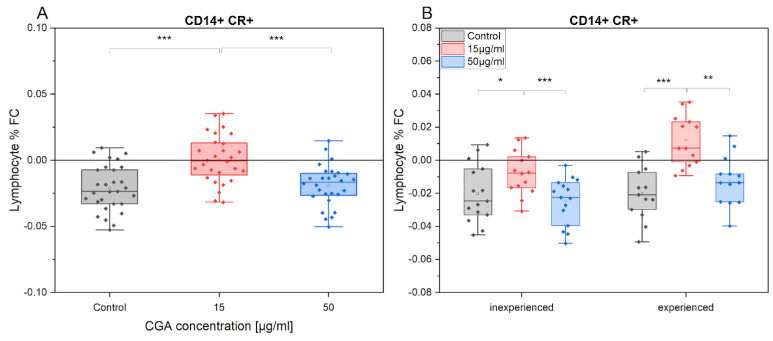
The fold change (FC) in CR (CellRox) intensity of CD14+ percentage gated from total lymphocytes/monocytes in response to exercise (**A**) in the absence and presence of CGA at 15 µg/mL and 50 µg/mL concentrations and at different horses’ advancement level (inexperienced, experienced) (**B**). Each dot represents one horse sample in a particular treatment condition, and means ± SEM (standard error of the mean) are presented. Significance levels are: * *p* < 0.05, ** *p* < 0.01, and *** *p* < 0.001.

The impact of training had a lesser effect on oxidative stress in CD14+ monocytes compared to CD5+ lymphocytes (FC = −0.02). Moreover, treatment with CGA at 15 µg/mL did not appear to elicit changes in ROS production in CD14+ cells. However, this significantly differed from the control treatment and the 50 µg/mL CGA treatment (control: *p* = 7.2 × 10^−6^, 50 µg/mL: *p* = 3.5 × 10^−5^) (Figure 7). These trends have also been observed in both groups of horses with varying levels of advancement. It is worth noting, that 15 µg/mL CGA treatment led to a slight increase in ROS production after training (FC = 0.01).

**Figure 8:**
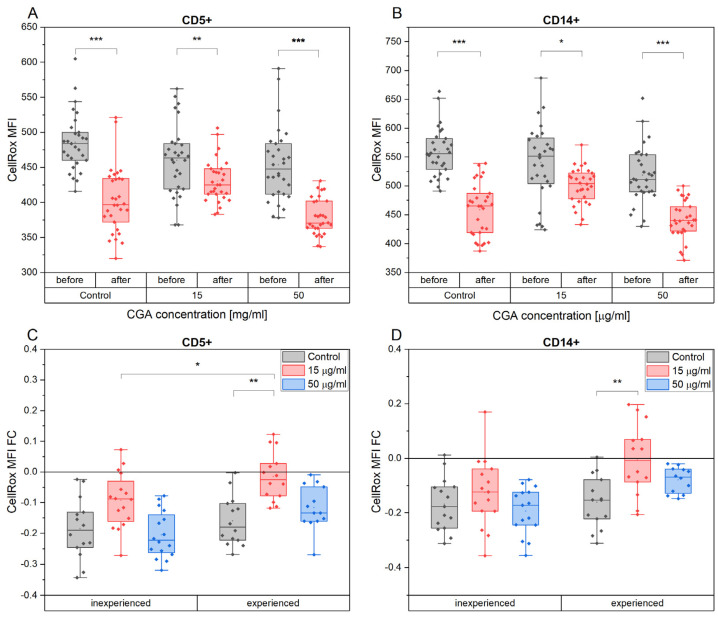
The ROS accumulation quantity expressed as CellRox mean fluorescence intensity (MFI) of CD5+ (**A**) and CD14+ (**B**) cell subtypes and the fold change (FC) in mean fluorescence intensity (MFI) of CD5+ (**C**) and CD14+ (**D**) in the two horses’ advancement groups, inexperienced and experienced, in response to the absence and presence of CGA at 15 µg/mL and 50 µg/mL concentrations. Each dot represents one individual horse’s sample in a particular treatment condition, and means ± SEM (standard error of the mean) are presented. Significance levels are * *p* < 0.05, ** *p* < 0.01, and *** *p* < 0.001.

After exercise, a noticeable decrease in ROS accumulation was observed in both CD5+ lymphocytes and CD14+ monocytes, regardless of CGA treatment (Figure 8A,B). However, among the various CGA-treated cell groups, those exposed to a concentration of 15 µg/mL displayed the least pronounced changes.

The extent of ROS accumulation changed in CD5+ lymphocytes, expressed as fold change (FC) in response to exercise, exhibited a lesser reduction in cells derived from the more experienced group of horses compared to the inexperienced group (Figure 8C). Notably, this difference reached statistical significance in cells treated with 15 µg/mL CGA (*p* = 0.049) (Figure 8C).

In the group of inexperienced horses’ cells, CGA treatment did not influence the post-exercise alterations in ROS accumulation for both CD5+ and CD14+ cell subtypes. However, within the experienced group, cells treated with 15 µg/mL CGA showed a subtle reduction in ROS accumulation after exercise (mean FC: CD5+ = −0.015 and CD14+ = −0.0056) in contrast to a more pronounced reduction in the control group (mean FC: CD5+ = −0.16 and CD14+ = −0.16) (CD5+: *p* = 0.0036; CD14+: *p* = 0.0088) (Figure 8D).

### 3.5. Chlorogenic Acid Effect on PBMCs Cytokine Production after Training

**Figure 9:**
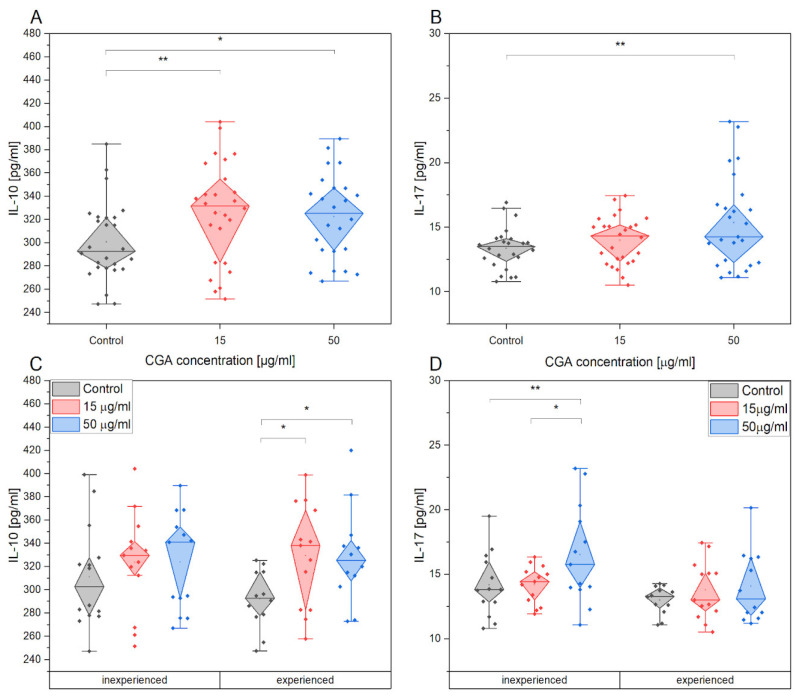
The change in IL-10 (**A**) and IL-17 (**B**) secretion in the absence and presence of CGA at 15 µg/mL and 50 µg/mL concentrations and its comparison between groups of experienced and inexperienced horses during exercise (**C**,**D**). Each dot represents one horse sample in a particular treatment condition, and means ± SEM (standard error of the mean) are presented. Significance levels are: * *p* < 0.05, ** *p* < 0.01.

The production of pro-inflammatory cytokines, including IL-1β, IL-8, INF-γ, TNF-α, and IL-17 as well as anti-inflammatory cytokine IL-10, was investigated in PBMCs collected after the training session (Figure 9). The supernatant was analyzed for cytokine levels. No significant changes were observed in the production of pro-inflammatory cytokines following CGA treatment. However, CGA treatment had a significant impact on the production of anti-inflammatory cytokines. Our study revealed an increase in IL-10 secretion compared to the control treatment, regardless of the concentration of CGA used. Additionally, IL-17 secretion was significantly higher in cells treated with 50 µg/mL CGA compared to the control treatment.

IL-10 is upregulated significantly after CGA stimulation in PBMCs of experienced horses, while IL-17 is upregulated in inexperienced horses.

## 4. Discussion

Our findings provide the first confirmation of the immunomodulatory function of CGA on PBMCs in racehorses. Previous studies conducted on humans have established the anti-inflammatory and anti-oxidative effects of CGA supplementation, positioning it as a therapeutic agent with diverse applications [7,49,50]. Given the significant physiological similarity between horses and humans in the field of exercise immunology [37,38,39,47], the addition of CGA to the equine diet holds the potential to offer various benefits to equine athletes.

### 4.1. Lymphocyte Phenotype and Proliferation

T lymphocytes play a critical role in the cellular immune system. The CD4+/CD8+ ratio is defined as the ratio of helper T cells (surface marker CD4) to cytotoxic T cells (surface marker CD8), where CD8+ cells are pro-inflammatory and CD4+ cells are anti-inflammatory. Recent studies have found that CGA may affect both the population of T cells and their proliferation. In human studies, it was confirmed that CGA at a concentration of 400 mg/kg improves cellular immunity by enhancing CD4+/CD8+ proliferation by decreasing the number of CD8+ T cells while at the same time increasing CD4+ T cell proliferation in a dose-dependent manner [51]. Similar findings regarding CD4+ lymphocytes were confirmed in our study.

In addition, several studies confirmed that CGA treatment reveals a significant role in regulating the CD4+/CD8+ ratio through the activation of CD4+ T cells and in stimulating T helper cells, which may directly contribute to the regulation of inflammation [52,53]. Reaching out to another publication, it was shown that CGA exhibits its anti-cancer benefits in breast cancer treatment by increasing the percentage of CD4+ and CD8+ T cells in the spleens of experimental mice [54]. It has been clarified that CGA can activate CD4 T cells by suppressing Toll-like receptor (TLR) 4 signaling molecules, including TLR4, p-IRAK1, p-IκB, and p-p38 [55]. Our results show that the presence of CGA does not affect the percentage of CD4+ and CD8+ cells, although it influences CD4+ proliferation in a dose-dependent manner. However, the lack of significant differences in the percent of T lymphocytes after CGA and exercise may be related to the fact that intense physical activity may inhibit the proliferation of CD4+ and CD8+ cells in humans as well as equine athletes [47,55]. It was concluded that those T cells tend to show a reduced response to mitogens and antigen-specific stimulation during intense exercise.

An additional objective of our research was to show how CGA affected CD4+FoxP3+ and CD8+FoxP3+ T-cell populations. FOXP3, which encodes a transcriptional repressor protein, is primarily expressed by CD4+ or CD8+ innate regulatory cells, ensuring immunological homeostasis and self-tolerance [55,56]. CGA administration at a dose of 50 μg/mL generates a significant increase in the percent of CD4 + FOXP3 + and CD8 + FOXP3 + T cells, according to our findings. It is worth noting that a significant difference was seen when comparing the maximum concentration (50 μg/mL) with the 15 μg/mL treatment, indicating substantial activation of CD8+FoxP3+ (*p* = 0.047). As CD4+FoxP3+ and CD8+FoxP3+ have strong immunoregulating activity [57,58], we confirmed that CGA may regulate the immune system response during exercise. Also, in response to exercise, the administration of CGA therapy increases the T reg population. The available data obtained on humans are consistent with our results [51,53].

### 4.2. ROS Production by T Lymphocytes and Monocytes

ROS develop naturally as byproducts of normal oxygen metabolism and are crucial intracellular signaling molecules [59]. Mitochondrial ROS tends to activate and regulate the development of Th17 and Th1 lymphocytes, and their low levels trigger the immunoregulatory enzyme indoleamine-2,3-dioxygenase and increase the activity of T reg lymphocytes. It has been proven that ROS plays a pivotal role as a T cell receptor (TCR) signaling molecule in various aspects of T lymphocyte-mediated immunity encompassing T cell proliferation, effector function, and apoptosis [60]. Moreover, ROS production by monocytes serves as a key machinery in innate immunity and inflammation [61]. Despite its critical immunostimulatory role, excess ROS can react with and damage biomolecules such as proteins, lipids, and DNA, which can disrupt redox equilibrium. Increased exposure to ROS leads to reduced phosphorylation and activation of NF-B, resulting in hyporeactivity of T lymphocytes. It is well-studied that excessive ROS production is triggered within the contracting muscle during physical exercise [62]. Therefore, it is essential to consider the importance of maintaining a balance between ROS and antioxidant proteins within the cell, especially during an intensive training regimen.

This balance plays a critical role in preserving the integrity of cell-mediated immunity. Several studies have shown that CGA has the biological ability to reduce ROS levels in a variety of concentrations, including 250 M CGA [63], 125 and 250 g/mL [64], 64 g/mL [65], 50 mg/mL [66], and 10 M [67]. This aligns with the results of our work. We confirmed that CGA significantly reduces the percentage of ROS-positive T lymphocytes CD5+ and CD14+ monocytes in racehorses. This effect is most pronounced at the highest CGA concentration tested compared to the control treatment (CD5+: *p* = 3.1 × 10^−5^, CD14+: *p* = 1.34 × 10^−6^).

In addition, in this study, it has been noticed that the important balance in ROS production, in response to exercise, can be preserved by the CGA treatment. Studies report that physical exercise induces upregulation of antioxidant enzyme pathways and thus reduces the oxidative state in skeletal muscles, liver, and heart [68,69]. We have observed a lowered percentage of CD5+ and CD14+ cells positive for ROS production after exercise. A uniform decrease in ROS generation is observed in both horse performance level groups, which is in contrast to our previous study [47]. However, in a previous study, tert-butyl hydroperoxide solution (TBHP) was used as an inducer of reactive oxygen species (ROS) production in all samples. In this study, we studied ROS production without additional stimulation besides exercise.

ROS generation plays an important role in muscle remodeling, and hypertrophy, through stimulating molecular pathways via proteins, including peroxisome proliferator-activated receptor-c coactivator (PGC1-α) and mitogen-activated protein kinases (MAPK) [70]. However, when cells were treated with 15 µg/mL of CGA, ROS production appeared to remain unchanged in post-exercise cells compared to pre-exercise levels. Notably, this observation is limited to the experienced group of horses, suggesting that CGA may enhance the positive adaptation specifically in the experienced individuals. ROS contributes to the maintenance of cartilage homeostasis and regulates apoptosis, extracellular matrix synthesis and breakdown [71,72,73], and cytokine production in chondrocytes [63], which is stronger in horses starting their training. Comparing CGA effects over PBMCs, we show that training has less effect on oxidative stress in CD14+ monocytes compared to CD5+ T lymphocytes. In our previous study, exercise had only an effect on ROS accumulation in CD14+ cells [47]. The ability of CGA to modulate ROS production is particularly important since low levels of ROS play an important role in controlling cellular functions by maintaining cellular antioxidant systems against oxidative stress as well as cytokine production. In summary, under physiological conditions, the equilibrium between ROS and antioxidant systems guarantees the proper functioning of T cells and the mounting of a controlled immune response.

### 4.3. Cytokines

CGA is known to promote an anti-inflammatory state by decreasing the level of the pro-inflammatory cytokine, as has been described in in vitro and in vivo animal and human studies [20,74,75,76]. The IL-8 diminished production has been observed in Caco-2 cells treated with 0.5–2 mM CGA [20]. Other studies confirmed that CGA, in a dose-dependent manner (2 to 20 μM), decreases the production of pro-inflammatory cytokines, mainly TNF-α, IL-8, IL-6, and IL-1β in vivo [20,74,75,76]. CGA’s ability to modulate cytokine secretion is believed to be associated with its capacity to reduce oxidative stress as it was mentioned earlier. This relationship has also been observed in our study, where CGA was found to decrease ROS production in immune cells. It has been suggested that by reducing oxidative stress, CGA can inhibit the activation of the NF-κB signaling pathway, leading to a decrease in the production of pro-inflammatory cytokines and other cellular mediators.

Indirect confirmation for that mechanism, despite the evident effect of reduced ROS production, is the increased production of IL-10 after CGA stimulation by PBMCs collected post-exercise. IL-10 exhibits diverse biological effects across different cell types [77], and it is the strongest anti-inflammatory cytokine. Its action promotes the downregulation of pro-inflammatory cytokines and Th1 lymphocyte functioning [78]. The observed elevation in CGA-induced IL-10 secretion after exercise could explain the subdued inflammatory response noted in our assessment of lymphocyte and monocyte activity changes from pre- to post-exercise cells. Remarkably, our study demonstrates that the anti-inflammatory effects of CGA are particularly pronounced in PBMCs from the well-trained group of horses, which is consistent with our previous findings. It is worth noting that the elevated IL-10 level is characteristic of progressing adaptation to exercise [47]. CGA stimulation might have been responsible for the further promotion of positive adaptation to training.

IL-17 is the only investigated pro-inflammatory cytokine that responds to CGA treatment. In fact, it has been confirmed that IL-17 is one of the highly enriched molecular pathways by CGA [79]. The CGA stimulation increases IL-17 secretion but only in post-exercise PBMCs of inexperienced horses. IL-17 is recognized for its ability to induce the production of various other pro-inflammatory factors. Through its action on endothelial cells, this cytokine facilitates the migration of neutrophils into inflamed tissues. The acute bout of exercise, as untrained horses experienced, might provoke the muscle damage inflammatory response, and it is possible that CGA treatment further promotes the IL-17 secretion by stimulating the CD4+ activity as was also confirmed in our study [80]. This thesis may be forced by the stimulation of IL-17 synthesis by PBMCs after exercise in untrained racehorses, which was confirmed in our previous study [47]. As was mentioned in our previous study, the inflammatory process and tendon remodeling are stimulated by IL-17 production not only by local tissues but also by PBMCs and it is balanced mainly by IL-10 upregulation [81]. IL-17 production is needed; however, its upregulation may not always be beneficial, especially in inexperienced horses. Thus, it confirms that CGA has a strong immunomodulatory effect and can be beneficial to horses at different fitness levels.

## 5. Limitations

The limitations of this study are the relatively low sample size (n = 29) and diversity of the horses as only two breeds were evaluated. On the other hand, Thoroughbreds and Arabians are the most popular racing breeds. In addition, the study was not performed in vivo; however, this was decided based on good ethical practices in performing experiments in animal models. The long-term effects and safety of CGA supplementation remain ambiguous, and its interactions with other common supplements or medications given to racehorses are unexplored. It should be noted that the effects of varying exercise intensities, durations, and other environmental factors influencing immune responses and ROS production could also impact the results.

## 6. Conclusions

The research presented here holds significant value for harnessing CGA’s potential as a nutritional supplementation to enhance the training performance of racehorses. The demonstrated immunomodulatory and oxidative stress reduction properties of this phenolic acid on PBMCs may greatly benefit athletes’ performance and recovery. The CGA anti-inflammatory effect is based on the stimulation of CD4+ proliferation and the immunophenotype of CD4+FoxP3+ and CD8+FoxP3+ regulatory T cells as well as IL-10 production. By understanding these effects, CGA could prove to be an asset in optimizing athletic performance and aiding in post-injury recovery. Thus, our findings have great meaning for future research or may have some practical applications for producing supplements for equine athletes. To fortify the study, it might be beneficial to delve into a long-term analysis of CGA’s effects, understand its interactions with other supplements, and assess its performance under diverse exercise regimens. Investigating the molecular mechanisms through which CGA operates, its impact on other equine cells beyond PBMCs, and linking the cellular benefits to behavioral or performance outcomes in horses could provide a more holistic view of CGA’s potential benefits in equine health.

## Figures and Tables

**Table 1:** Monoclonal antibodies employed to tag peripheral blood mononuclear cells (PBMCs) for flow cytometry labeling.

Antibody	Clone; Dilution	Source	Target Cell
CD4:PE	CVS4; 1:10	BioRad, Berkeley, CA, USA	Lymphocytes
CD8:FITC	CVS21; 1:10	BioRad, Berkeley, CA, USA	Lymphocytes
CD5:PE	CVS5; 1:10	BioRad, Berkeley, CA, USA	Lymphocytes
CD14:AF405	433423; 1:10	R&D Systems, Minneapolis, MI, USA	Monocytes
MHCII:FITC	CVS20; 1:20	BioRad, Berkeley, CA, USA	Monocytes
FoxP3:APC	FJK-16s; 1:10	Life Technologies, Bleiswijk, The Netherland	Lymphocytes

## Data Availability

All datasets generated and/or analyzed during the current study are presented in the article, the accompanying SourceData (https://doi.org/10.18150/LAVBG5, RepOD, V1) or Supplementary Information files, are available from the corresponding author upon reasonable request.

## Supplementary Materials

The following supporting information can be downloaded at: https://www.mdpi.com/article/10.3390/antiox12111924/s1, Figure S1: CD4+ count fold change (FC) in response to exercise (A) at different advancement levels (B) in the absence and presence of CGA at 15 µg/mL and 50 µg/mL concentrations. Each dot represents one horse sample in a particular treatment condition, and means ± SEM (standard error of the mean) are presented. Significance levels are: * *p* < 0.05, ** *p* < 0.01, and *** *p* < 0.001; Figure S2: Presentation of CD8+ count fold change (FC) in response to exercise in general (A) and at different advancement levels (B) in the absence and presence of CGA at 15 µg/mL and 50 µg/mL concentrations. Each dot represents one horse sample in a particular treatment condition, and means ± SEM (standard error of the mean) are presented. Significance levels are: * *p* < 0.05, ** *p* < 0.01, and *** *p* < 0.001; Figure S3: Percentages of positive cells: CD14+MHCII−, CD14−MHCII+, CD14−MHCII−, and CD14+MHCII+ gated from total monocytes (A), and its FC in response to exercise (B) in the absence and presence of CGA at 15 µg/mL and 50 µg/mL concentrations. Each dot represents one horse sample in a particular treatment condition, and means ± SEM (standard error of the mean) are presented. Significance levels are: * *p* < 0.05, ** *p* < 0.01, and *** *p* < 0.001; Figure S4: Presentation of CD14+MHCII+ count fold change (FC) in response to exercise in general (A) and at different advancement levels (B) in the absence and presence of CGA at 15 µg/mL and 50 µg/mL concentrations. Each dot represents one horse sample in a particular treatment condition, and means ± SEM (standard error of the mean) are presented. Significance levels are: * *p* < 0.05, ** *p* < 0.01, and *** *p* < 0.001; Figure S5: Presentation of CD14+MHCII− count fold change (FC) in response to exercise in general (A) and at different advancement levels (B) in the absence and presence of CGA at 15 µg/mL and 50 µg/mL concentrations. Each dot represents one horse sample in a particular treatment condition, and means ± SEM (standard error of the mean) are presented. Significance levels are: * *p* < 0.05, ** *p* < 0.01, and *** *p* < 0.001; Figure S6: Presentation of CD14−MHCII+ count fold change (FC) in response to exercise in general (A) and at different advancement levels (B) in the absence and presence of CGA at 15 µg/mL and 50 µg/mL concentrations. Each dot represents one horse sample in a particular treatment condition, and means ± SEM (standard error of the mean) are presented. Significance levels are: * *p* < 0.05, ** *p* < 0.01, and *** *p* < 0.001; Figure S7: Presentation of CD14−MHCII− count fold change (FC) in response to exercise in general (A) and at different advancement levels (B) in the absence and presence of CGA at 15 µg/mL and 50 µg/mL concentrations. Each dot represents one horse sample in a particular treatment condition, and means ± SEM (standard error of the mean) are presented. Significance levels are: * *p* < 0.05, ** *p* < 0.01, and *** *p* < 0.001.
