# Supplementary material for: Impact of Chlorogenic Acid on Peripheral Blood Mononuclear Cell Proliferation, Oxidative Stress, and Inflammatory Responses in Racehorses during Exercise
Source: Antioxidants (Basel). 2023 Oct 28;12(11):1924. doi: 10.3390/antiox12111924 (PMC10669817; doi:10.3390/antiox12111924)
Supplement: Supplementary file 1 [file antioxidants-12-01924-s001.zip › antioxidants-2576003-supplementary.pdf]

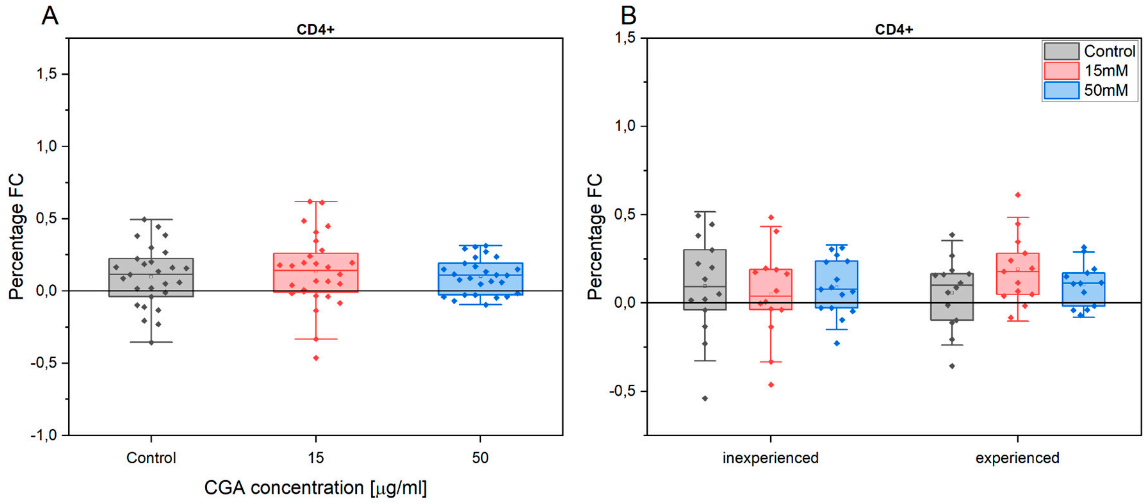

Figure S1. CD4+ count fold change (FC) in response to exercise (A) at different advancement levels (B) in the absence and presence of CGA at 15µg/ml and 50µg/ml concentrations. Each dot represents one horse sample in a particular treatment condition and means ± SEM (standard error of the mean) are presented. Significance levels are: * $p < 0.05$, ** $p < 0,01$, *** $p < 0,001$

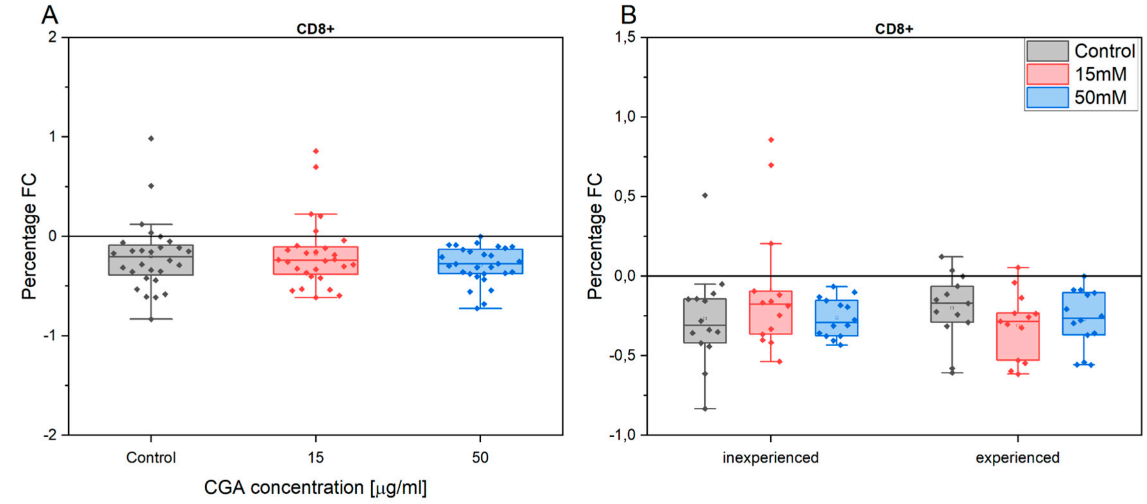

Figure S2. Presentation of CD8+ count fold change (FC) in response to exercise in general, (A) and at different advancement levels (B) in the absence and presence of CGA at 15µg/ml and 50µg/ml concentrations. Each dot represents one horse sample in a particular treatment condition, and means ± SEM (standard error of the mean) are presented. Significance levels are: * $p < 0.05$, ** $p < 0,01$, *** $p < 0,001$

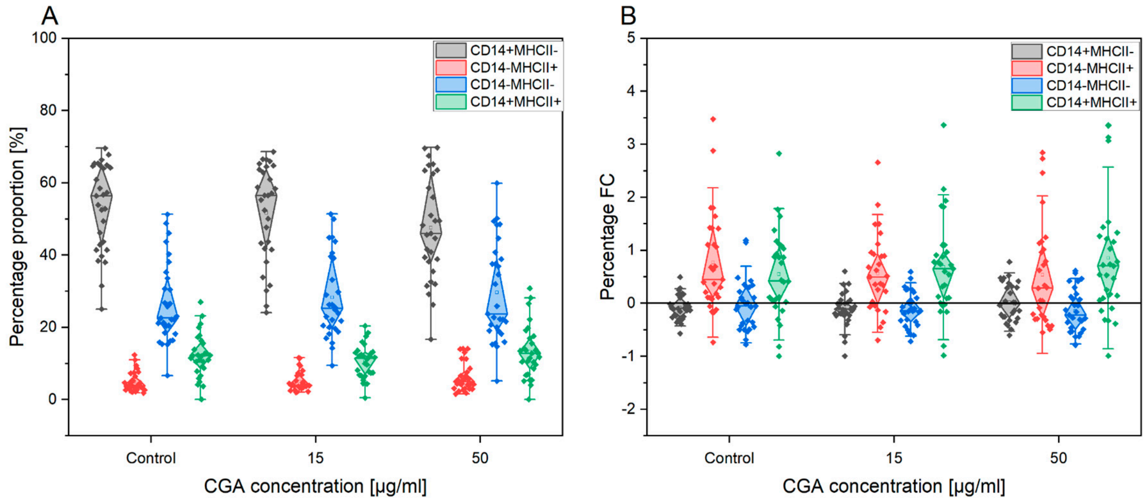

Figure S3. Percentages of positive cells: CD14+MHCII−, CD14-MHCII+, CD14-MHCII- and CD14+MHCII+ gated from total monocytes (A), and its FC in response to exercise (B) in the absence and presence of CGA at 15µg/ml and 50µg/ml concentrations. Each dot represents one horse sample in a particular treatment condition and means ± SEM (standard error of the mean) are presented. Significance levels are: * p < 0.05, ** p < 0,01, *** p < 0,001

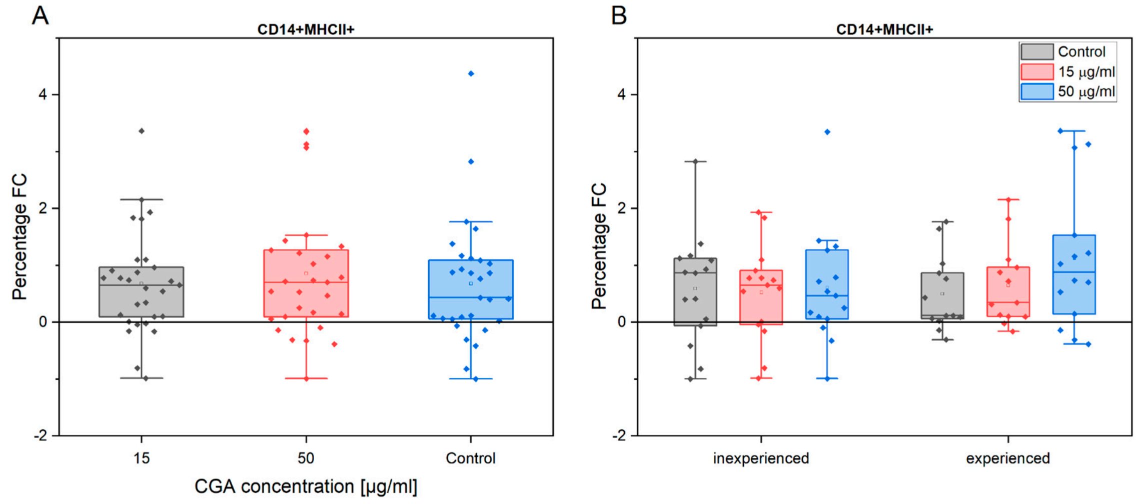

Figure S4. Presentation of CD14+MHCII+ count fold change (FC) in response to exercise in general, (A) and at different advancement levels (B) in the absence and presence of CGA at 15µg/ml and 50µg/ml concentrations.Each dot represents one horse sample in a particular treatment condition, and means ± SEM (standard error of the mean) are presented. Significance levels are: * p < 0.05, ** p < 0,01, *** p < 0,001

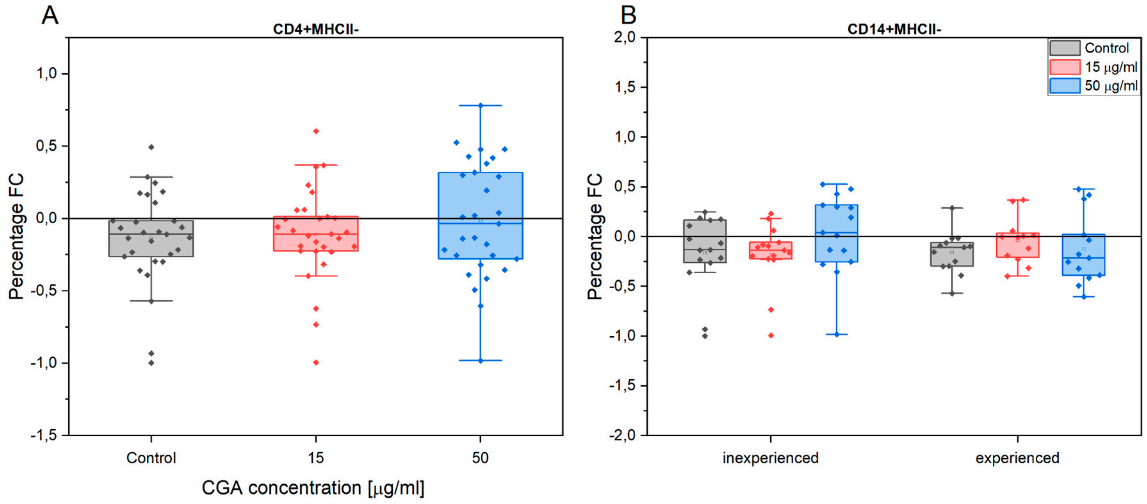

Figure S5. Presentation of CD14+MHCII- count fold change (FC) in response to exercise in general, (A) and at different advancement levels (B) in the absence and presence of CGA at 15µg/ml and 50µg/ml concentrations. Each dot represents one horse sample in a particular treatment condition and means ± SEM (standard error of the mean) are presented. Significance levels are: * $p < 0.05$, ** $p < 0,01$, *** $p < 0,001$

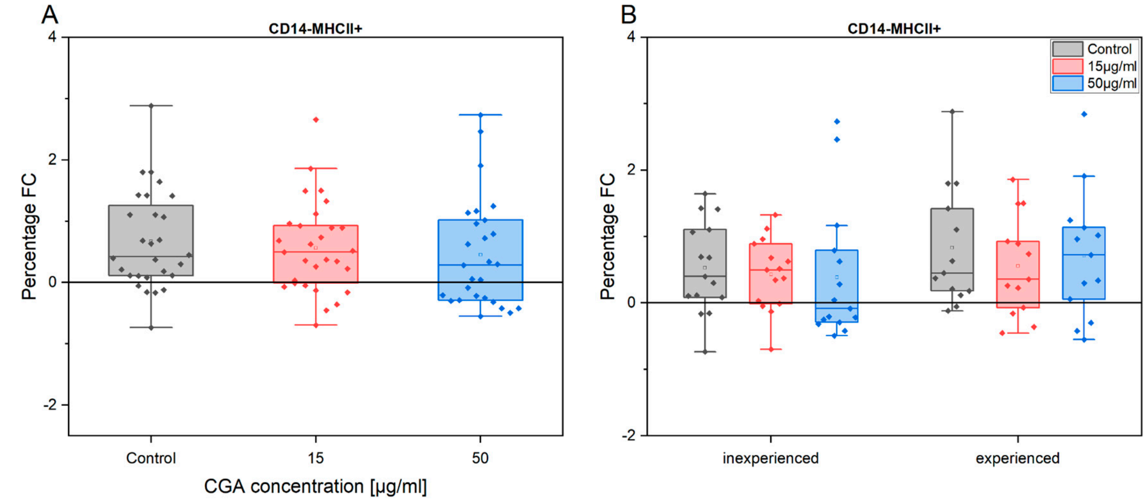

Figure S6. Presentation of CD14-MHCII+ count fold change (FC) in response to exercise in general, (A) and at different advancement levels (B) in the absence and presence of CGA at 15µg/ml and 50µg/ml concentrations. Each dot represents one horse sample in a particular treatment condition and means ± SEM (standard error of the mean) are presented. Significance levels are: * $p < 0.05$, ** $p < 0,01$, *** $p < 0,001$

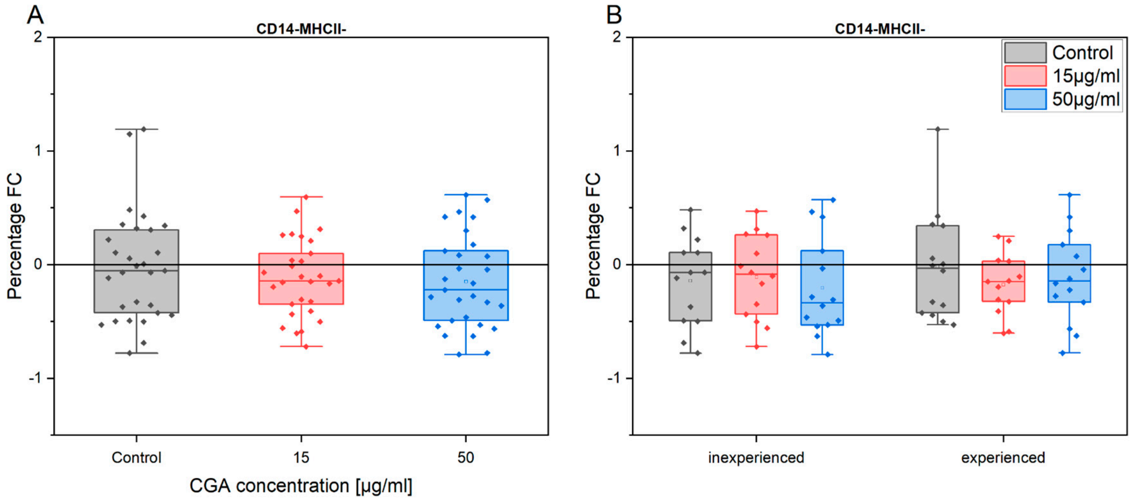

Figure S7. Presentation of CD14-MHCII- count fold change (FC) in response to exercise in general, (A) and at different advancement levels (B) in the absence and presence of CGA at 15µg/ml and 50µg/ml concentrations. Each dot represents one horse sample in a particular treatment condition, and means ± SEM (standard error of the mean) are presented. Significance levels are: * $p < 0.05$, ** $p < 0{,}01$, *** $p < 0{,}001$
